# IL‐8 promotes cell migration through regulating EMT by activating the Wnt/β‐catenin pathway in ovarian cancer

**DOI:** 10.1111/jcmm.14848

**Published:** 2019-12-02

**Authors:** Jirui Wen, Zhiwei Zhao, Liwei Huang, Ling Wang, Yali Miao, Jiang Wu

**Affiliations:** ^1^ Deep Underground Space Medical Center West China Hospital Sichuan University Chengdu China; ^2^ West China School of Basic Medical Sciences & Forensic Medicine Sichuan University Chengdu China; ^3^ West China School of Stomatology Medicine Sichuan University Chengdu China; ^4^ Department of Obstetrics and Gynecology Key Laboratory of Birth Defects and Related Diseases of Women and Children of MOE West China Second University Hospital Sichuan University Chengdu China

**Keywords:** epithelial‐mesenchymal transition, interleukin‐8, migration, ovarian cancer, Wnt/β‐catenin pathway

## Abstract

Interleukin‐8 (IL‐8), as an inflammatory chemokine, has been previously shown to contribute to tumorigenesis in several malignancies including the ovarian cancer. However, little is known about how IL‐8 promotes the metastasis and invasion of ovarian cancers cells. In this study, we found that IL‐8 and its receptors CXCR1 and CXCR2 were up‐regulated in advanced ovarian serous cancer tissues. Furthermore, the level of IL‐8 and its receptors CXCR1 and CXCR2 expression were associated with ovarian cancer stage, grade and lymph node metastasis. In vitro, IL‐8 promoted ovarian cancer cell migration, initiated the epithelial‐mesenchymal transition (EMT) program and activated Wnt/β‐catenin signalling. However, when treated with Reparixin (inhibitor of both IL‐8 receptors CXCR1 and CXCR2), effect of both endogenous and exogenous IL‐8 was reversed. Together, our results indicated that IL‐8 triggered ovarian cancer cells migration partly through Wnt/β‐catenin pathway mediated EMT, and IL‐8 may be an important molecule in the invasion and metastasis of ovarian cancer.

## 1 INTRODUCTION

Ovarian cancer is a disease that has the highest mortality rate among all gynaecological malignancies. It was estimated that 22 440 new cases and 14 080 deaths of ovarian cancer are expected in American in 2018, in the meanwhile a 5‐year survival rate that can be as low as 30% when diagnosed after the cancer.^1^ The development of ovarian cancer begins at the ovary and is more apt to through peritoneal metastasis to adjacent organs.^2^ Even with the use of recommended therapies, patients diagnosed with ovarian cancer always have the high rates of relapse because the disseminating ovarian cancer cells form the secondary tumour foci in the peritoneal cavity.^3^, ^4^ Ovarian tumour microenvironment is regarded as a place rich in proinflammatory cytokines and chemokines, and this proinflammatory microenvironment is considered to be a prominent factor affecting the spread of ovarian cancer cells.^5^

Physiological environment is determined by many chemical factors which direct influence tissue function and also have a great impact on diseases such as cancer. Inflammation mediators play an essential role in ovarian cancer pathogenesis, affecting ovarian cancer initiation, promotion and progression.^6^ In the tumour microenvironment, related chemokines and cytokines promote the angiogenesis of tumour tissues and the phenotype transformation of cancer cells, which in turn leads to tumour invasion and metastasis.^7^ In ovarian cancer, chemokines regulate the cancer cell survival, tumour angiogenesis, cancer cell proliferation and metastasis.^8^ Elucidating the relationship between chemokines and ovarian cancer may reveal new target therapies. Interleukin‐8 (IL‐8) is an inflammatory chemokine that mediates the activity of immune cells and chemotaxis, leading to chronic inflammation. The activity of IL‐8 is mainly determined by the binding of IL‐8 to its receptors CXCR1 and CXCR2.^9^ In ovarian cancer, increased secretion of IL‐8 has been proved to promote tumour growth and upgrade metastasis.^10^ However, how IL‐8 enhances the metastasis and invasion of ovarian cancers cells remains unknown.

Recent studies have suggested that epithelial‐mesenchymal transition (EMT) was closely related to the invasion and metastasis of ovarian cancer,^11^ which may provide the molecular basis for the IL‐8–triggered ovarian cancer cell migration. In the process of EMT in tumour cells, special markers of epithelial cells such as E‐cadherin are down‐regulated, causing a loss of the epithelial features and a acquire of some mesenchymal features.^12^ When cancer cells undergoing EMT, the tumour cells locally infiltrate and distantly migrate, which ultimately leads to a decrease in patient survival rate.^13^ In ovarian cancer, EMT is thought to be involved in the metastasis and be predicted of poor prognosis, the reduction in E‐cadherin on the cell surface is closely correlated with poor overall survival of patients with ovarian cancer.^14^

For the present study, we correlated the expression of IL‐8 and its receptors (CXCR1, CXCR2) with clinical measures (staging, tumour grade and overall survival) in patients with ovarian cancer. We also found the regulatory role of IL‐8–induced EMT in ovarian cell lines SKOV3 and A2780. Furthermore, our results indicated that IL‐8 may induce EMT of ovarian cancer cells partly through Wnt/β‑catenin signalling pathway. Therefore, the study revealed the significant roles and the potential mechanisms of IL‐8 in ovarian cancer migration and provided new therapeutic and prognostic targets.

## 2 MATERIALS AND METHODS

### 2.1 Specimen and immunohistochemistry (IHC)

All ovarian cancer specimens (n = 93) were obtained from surgical patients at the department of gynaecology, West China Second University Hospital (Chengdu, China). The patient was pathologically diagnosed with serous ovarian cancer and did not receive any other treatment prior to harvesting the specimen. All patients received written informed consent, which was approved by the Ethics Committee of Sichuan University. IHC was performed to detect IL‐8 and its receptors (CXCR1, CXCR2) with the using of anti‐IL‐8, anti‐CXCR1 and anti‐CXCR2 (1:100, Abcam) antibodies. Heat‐mediated antigen retrieval was performed in a pH 6.0 citrate buffer in a 98°C water bath for 15 minutes. Primary antibody was incubated at 4°C overnight, and secondary antibody was incubated at room temperature for 1 hours, followed by counterstaining with haematoxylin and visualization with the DAB. Finally, the sections were mounted using neutral gum. Two investigators independently assessed and scored the immunostaining based on staining intensity. Moderate and intense staining was defined as high expression, while no and weak staining was defined as low expression.

### 2.2 Bioinformatics analysis

We subjected the IL‐8, CXCR1 and CXCR2 to survival analysis using the online database (Gene Expression Profiling Interactive Analysis). *P*‐values were calculated with log‐rank (Mantel‐Cox) test. Patients were classified into ‘low’ and ‘high’ expression based on auto selected best cut‐off in the database.

### 2.3 Cell cultures

Two human ovarian cancer cell lines (SKOV3, A2780) were purchased from Nanjing KeyGen Biotech.Inc Cells were cultured in α‐MEM medium (Hyclone) supplemented with 10% fetal bovine serum (Israel) and 1% penicillin‐streptomycin (Hyclone), and every 3 days, the medium was changed. All cell cultures were maintained in a humidified incubator (Heraeus) with 5% CO_2_ saturation at 37°C, and the cells in their third or fourth passage were used.

To investigate whether IL‐8 played a key role in facilitating cell migration, the exogenous IL‐8 and the inhibitor of IL‐8 receptor (Reparixin) were used. Because the CXCR1/2 binds to IL8 with high affinity and transduces the signal through a G‐protein–activated second messenger system, the CXCR1/2 antagonism (Reparixin, MCE, USA) was commonly used to inhibit the IL‐8 signalling pathway. The cells were divided into four groups: cells without the exogenous IL‐8 or Reparixin but contain the endogenous IL‐8 (Endogenous IL‐8 was secreted by the ovarian cancer cells); cells treated with exogenous IL‐8 (100 ng/mL, MCE, USA) for 48 hours; cells treated with Reparixin (0.1 μmol/L) targeting CXCR1/2 for 48 hours to block the effect of endogenous and exogenous IL‐8; and cells treated with Reparixin (0.1 μmol/L) combined IL‐8 (100 ng/mL) for 48 hours.

### 2.4 Immunocytochemistry

The SKOV3 and A2780 cells were digested by trypsin‐EDTA and seeded into 24 cell plates. Then, the cells were incubated at 37°C, washed with PBS and fixed in 4.00% paraformaldehyde for 15 minutes. After permeablization using 0.50% Triton X‐100 and blocking by 5.00% normal goat serum, the cells were incubated with the using of anti–IL‐8, anti‐CXCR1 and anti‐CXCR2 (1:100, Abcam) antibodies at 4°C overnight, and the second antibody was incubated for 1 hours at room temperature. Finally, the cells were counterstained with haematoxylin and visualized with the DAB.

### 2.5 Monolayer wound healing assay

Migration ability was measured using the wound healing assay. SKOV3 and A2780 were grown in 6‐well plates. When the cells reached 80% density, a 100 μL pipette tip was used to create a horizontal line through the wells in the confluent cells, and then, the cells were incubated in fresh medium for 48 hours. Each experiment was repeated at least three times. The image was processed using ImageJ software (Rawak Software, Inc) and was calculated for the proportion of relative wound closure.

### 2.6 Transwell migration assay

Cell migration assay was performed using Transwell chambers. For this assay, cells were seeded in 100 μL of FBS‐free medium in the upper chamber of the well, while the lower chamber were filled with 600 μL of 20% FBS medium. Following incubation for 24 hours, the migrating cells on the bottom surface were fixed with methanol, stained with 0.1% crystal violet and imaged. The number of cells was calculated in at least five random regions of each chamber, and then, the average was calculated.

### 2.7 Immunofluorescence

The SKOV3 and A2780 cells cultured on glass slides were fixed with 4% paraformaldehyde for 20 minutes and then blocked with bovine serum albumin (BSA) at room temperature for 1 hours. Next, the cells were washed three times with 0.1% Triton X‐100/ PBS. Then, cells were incubated in F‐actin (1:100, Solarbio, China) for 2 hour and counterstained with DAPI (1:800, Solarbio, China) for 10 minutes. Finally, the cells were mounted on 50% glycerol/PBS and imaged using a Nikon eclipse Ti‐S microscope.

### 2.8 Western blot analysis

After the separation by sodium dodecyl sulphate‐polyacrylamide gel (SDS‑PAGE), the cellular protein was transferred to polyvinylidene fluoride (PVDF) membranes (Invitrogen Life Technologies). Followed the blocking with 5% fat‑free milk, the membrane was incubated with primary antibodies against E‑cadherin, vimentin, Wnt5a, p‐β‐catenin, β‐catenin, Met, C‐Jun and GAPDH (all from Cell Signaling Technology, Inc) overnight at 4°C and then incubated with a secondary antibody for 1 hours. Enhanced chemiluminescence kit (Beyotime Institute of Biotechnology) was used to visualize the blots.

### 2.9 Statistical analysis

All of the statistical analyses were performed with SPSS 18.0 software (SPSS Inc). Results were expressed as mean ± SD of at least three independent experiments performed. Treatment groups were compared using one‐way analysis of variance (ANOVA), and Student's *t* test. *P*‑values < .05 were considered to indicate a statistically significant difference.

## 3 RESULTS

### 3.1 IL‐8 and its receptors expression in ovarian cancer

**Figure 1:**
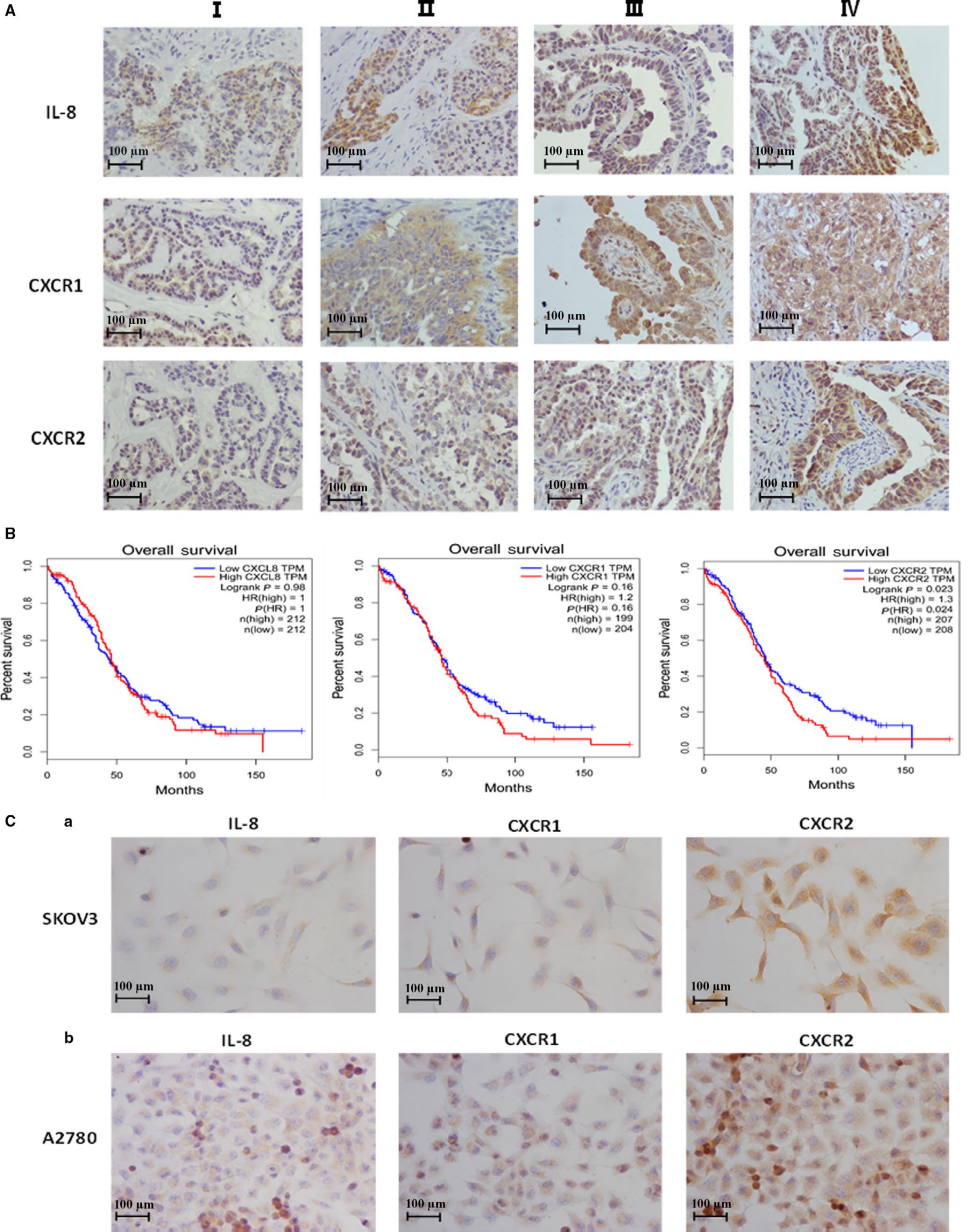
Expression of IL‐8, CXCR1 and CXCR2 in serous ovarian carcinomas and ovarian cancer cell lines. A, Representative IHC images showed the expression of IL‐8, CXCR1 and CXCR2 in serous ovarian cancer tissues of different stages (The brown part in the panels). IL‐8, CXCR1 and CXCR2 were highly expressed in III‐IV patients (n = 65), while lowly expressed in I‐II patients (n = 28). Scale bars = 100 μm. B, Correlation of the overall survival with the expression of IL‐8, CXCR1 and CXCR2 in GEPIA data set. *P*‐values were calculated with log‐rank (Mantel‐Cox) test. Reduced survival for ovarian cancer patients expressing the high levels of CXCR2 was found. C, Representative images showed immunocytochemistry staining for ovarian cancer cells demonstrating the expression of IL‐8, CXCR1 and CXCR2 in the cell membrane, cytoplasm and nucleus. The IL‐8, CXCR1 and CXCR2 were mainly expressed in the cell membrane and cytoplasm. Scale bars = 100 μm

To identify the expression of IL‐8, CXCR1 and CXCR2 in serous ovarian cancer, we collected patients with ovarian cancer from West China Second University Hospital. The clinical‐pathological characteristics of the case (age, tumour grade, distant metastasis and stage) and their relationship with the expression of IL‐8, CXCR1 and CXCR2 were reported in Table 1, and the high expression of IL‐8 and its receptors in high‐stage serous ovarian cancer were shown in Figure 1A. We found that the high expression of IL‐8 and CXCR2 were closely correlated with high tumour grade, tumour distant metastasis and high tumour stage, and the high expression of CXCR1 was closely related to high tumour grade and tumour distant metastasis. To further determine whether the IL‐8 and its receptors were associated with prognosis of patients with ovarian cancer, we subjected the IL‐8, CXCR1 and CXCR2 to survival analysis using the online database (Gene Expression Profiling Interactive Analysis). Data showed that CXCR2 was significantly associated with poor overall survival (OS) (Figure 1B). Thereby, the IL‐8 and its receptors, especially CXCR2, may be important mediators of ovarian cancer metastasis.

**Table 1:** Expression of IL‐8, CXCR1 and CXCR2 in serous ovarian carcinomas

Variable	N	IL‐8		*P* value	CXCR1		*P* value	CXCR2		*P* value
		Low	High		Low	High		Low	High	
Age										
≤60	69	28	41	.701	38	31	.140	37	32	.440
>60	24	8	16		18	6		10	14	
Differentiation										
Well	25	15	10	.005	14	11	.007	15	10	.004
Moderate	42	17	25		32	10		26	16	
Poor	26	4	22		10	16		6	20	
Distant metastasis										
Yes	22	2	20	.003	8	14	.018	6	16	.024
No	71	34	37		48	23		41	30	
Tumour stage										
I‐II	28	22	6	<.001	20	8	.223	19	9	.028
III‐IV	65	14	51		36	29		28	37	

Note

*P* value was calculated using one‐way analysis of variance (ANOVA).

### 3.2 Expression of IL‐8 and its receptors in ovarian cancer cell lines

To investigate the in vitro expression and sub‐cellular localization of IL‐8 and its receptors, the ovarian cancer cells were studied by immunocytochemistry. By studying the expression levels of IL‐8, CXCR1 and CXCR2 in different human ovarian cancer cell lines (SKOV3, A2780), we confirmed the expression of IL‐8 and its receptors in all cell lines tested (Figure 1C). Besides, cell membrane, cytoplasm and nucleus localization of IL‐8 and its receptors were observed in all cell lines. Notably, the IL‐8, CXCR1 and CXCR2 were mainly expressed in the cell membrane and cytoplasm. Therefore, the IL‐8 and its receptors could play their role in the ovarian cancer cells (SKOV3, A2780).

### 3.3 Inhibition of IL‐8 receptors attenuated migration of ovarian cancer cells

**Figure 2:**
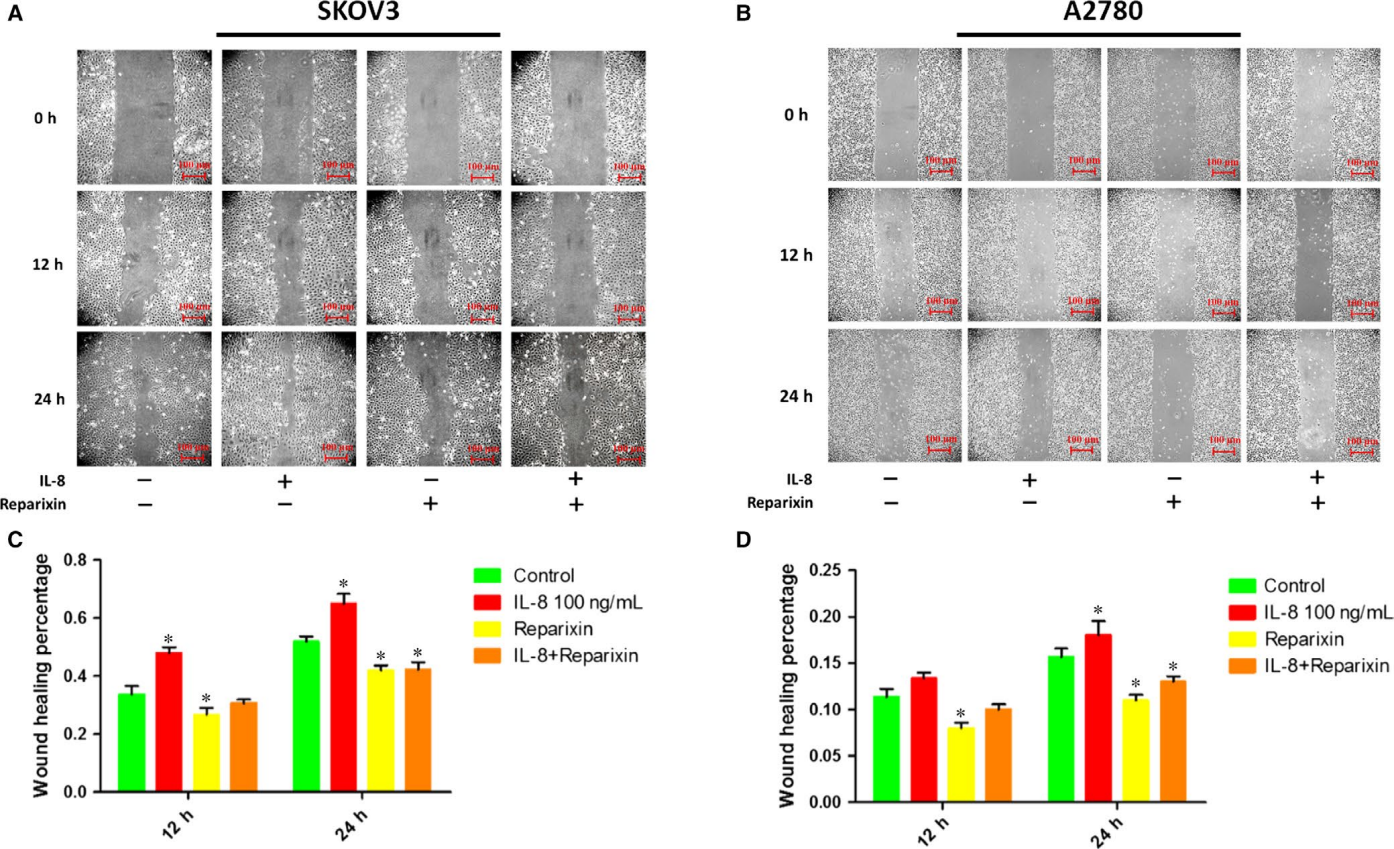
The effects of IL‐8 on the migration ability of ovarian cancer cells illustrated by the monolayer wound healing assay. Typical optical images illustrating the scratch injury wound of SKOV3 (A) and A2780 (B) at 0, 12 and 24 h, Scale bars = 100 μm. C, Statistical results of the wound healing percentage of SKOV3 at 12 and 24 h based on the scratch injury wound. D, Statistical results of the wound healing percentage of A2780 at 12 and 24 h based on the scratch injury wound. *P < .05 vs Control

**Figure 3:**
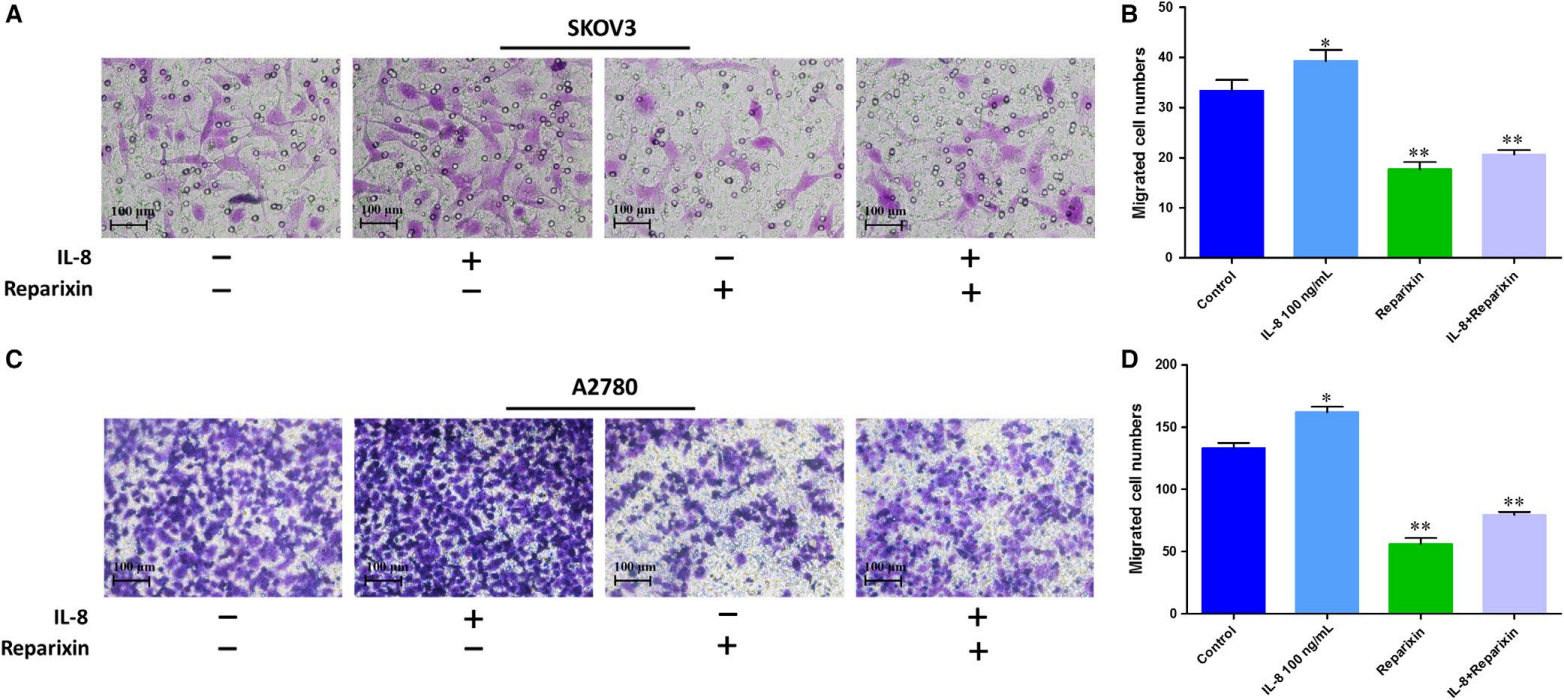
The effects of IL‐8 on the migration ability of ovarian cancer cells illustrated by the Transwell assay. Typical optical images of SKOV3 (A) and A2780 (C) illustrated cell migration at 24 h. The cells crossed through the pores of Transwell chamber were stained by crystal violet, Scale bars = 100 μm. B, Statistical results of the migrated SKOV3 cell number based on the Transwell assay. D, Statistical results of the migrated A2780 cell number based on the Transwell assay. *P < .05, **P < .01 vs Control

To investigate whether IL‐8 played a key role in facilitating cell migration, wound healing and Transwell assays were performed. As was shown in Figures 2 and 3, the wound healing percentage and the migrated cell number increased when treated with the exogenous IL‐8, which suggested that exogenous IL‐8 could promote the migration of ovarian cancer cells. However, when treated with the Reparixin (inhibitor of IL‐8 receptors CXCR1 and CXCR2), the migration of ovarian cancer cells decreased compared with the control group no matter whether treated or not treated with the IL‐8. Therefore, the Reparixin could significantly block the effect of endogenous and exogenous IL‐8.

### 3.4 Influence of IL‐8 on the cytoskeleton of ovarian cancer cells

**Figure 4:**
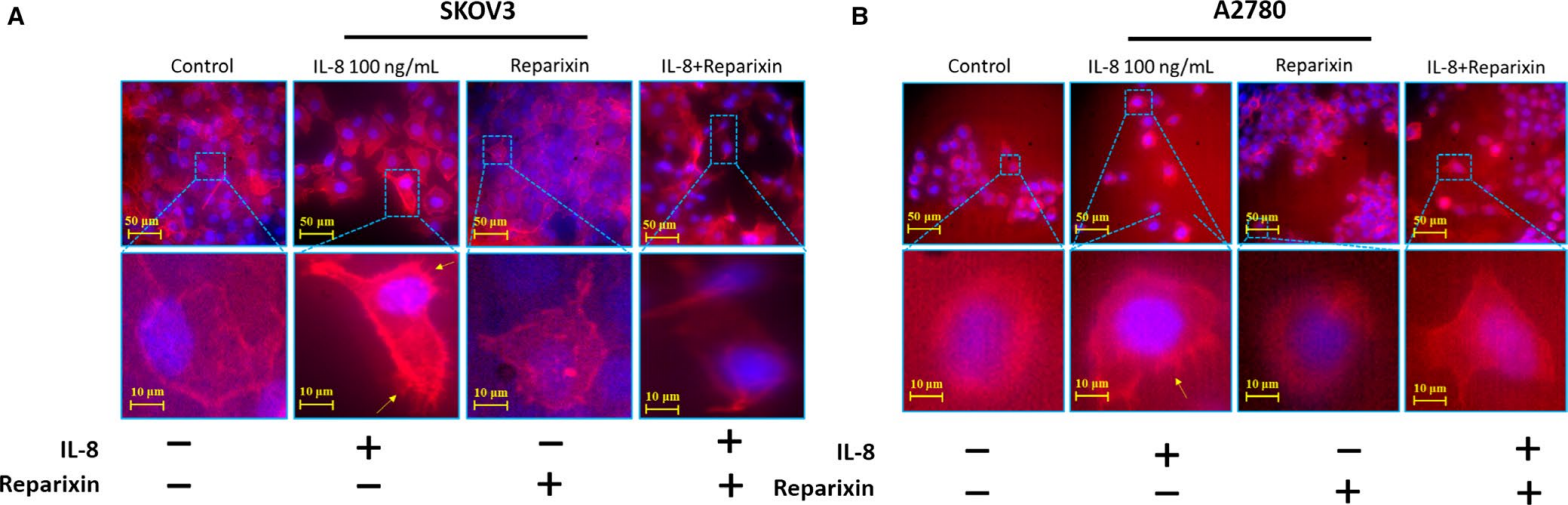
IL‐8 promote the cilia formation of SKOV3 cells (A) and A2780 cells (B). The images showed the cytoskeleton (red) and nucleus (blue). The amplified images of framed regions showed the location of filopodia. Scale bar = 10 μm. ↑, filopodia

To investigate the effect of IL‐8 on the cytoskeleton of ovarian cancer cells, F‐actin was detected by immunofluorescence assay. The phalloidin staining showed more protrusions formed when stimulated by the IL‐8, while the inhibitor of IL‐8 could significantly block the effect of endogenous and exogenous IL‐8 (Figure 4). This result suggested that the IL‐8 possibly rearranged cytoskeletal F‐actin assembly and promoted cellular filopodia, and ultimately retarded cellular motility.

### 3.5 IL‐8 induced EMT activation in ovarian cancer cells

**Figure 5:**
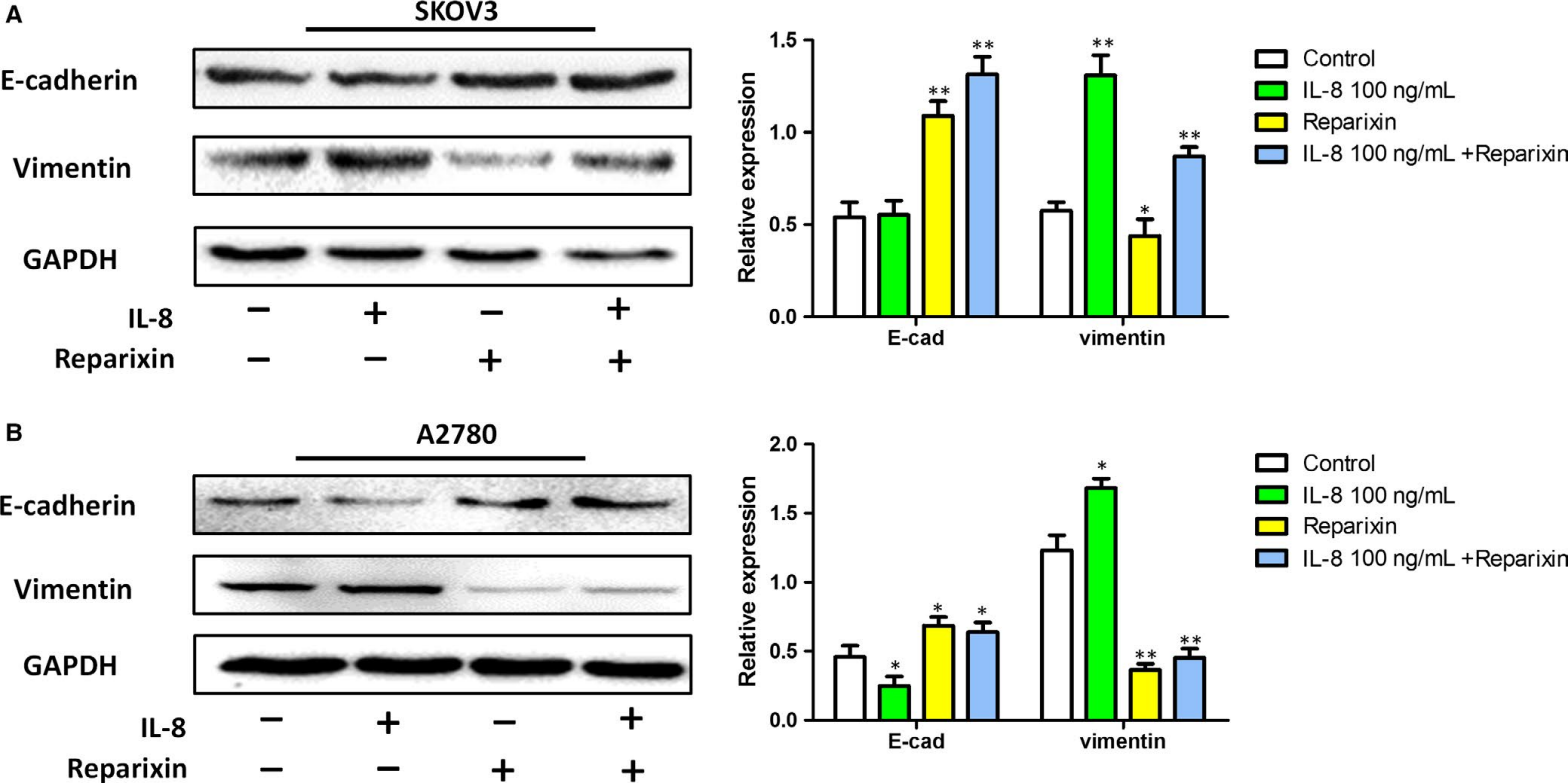
Expression of EMT biomarkers in ovarian cancer cells. A, The expression levels of EMT biomarkers in SKOV3 cells were quantified by image analysis of the Western blot bands. The expression of GAPDH in each group was taken as intrinsic controls, and relative expressions of each protein were calculated. B, The expression levels of EMT biomarkers in A2780 cells were quantified by image analysis of the Western blot bands. Values represented the Mean ± SD from three independent experiments. *P < .05, **P < .01 vs Control

To obtain further insights into the EMT‐promoting capability of IL‐8 in ovarian cancer, E‐cadherin and vimentin were detected using Western blotting. As shown in Figure 5, compared with the control, E‐cadherin level was markedly decreased and the vimentin level was markedly increased in exogenous IL‐8–treated group. However, treating with Reparixin in Reparixin group and Reparixin +IL‐8 group decreased vimentin level, as well as increased E‐cadherin level. The results revealed that treatment with IL‐8 led to an increased activity of EMT process. Moreover, when using inhibitor to block the effect of endogenous and exogenous IL‐8, the activity of EMT process decreased.

### 3.6 IL‐8 promoted EMT by up‐regulating the Wnt/β‐catenin pathway

**Figure 6:**
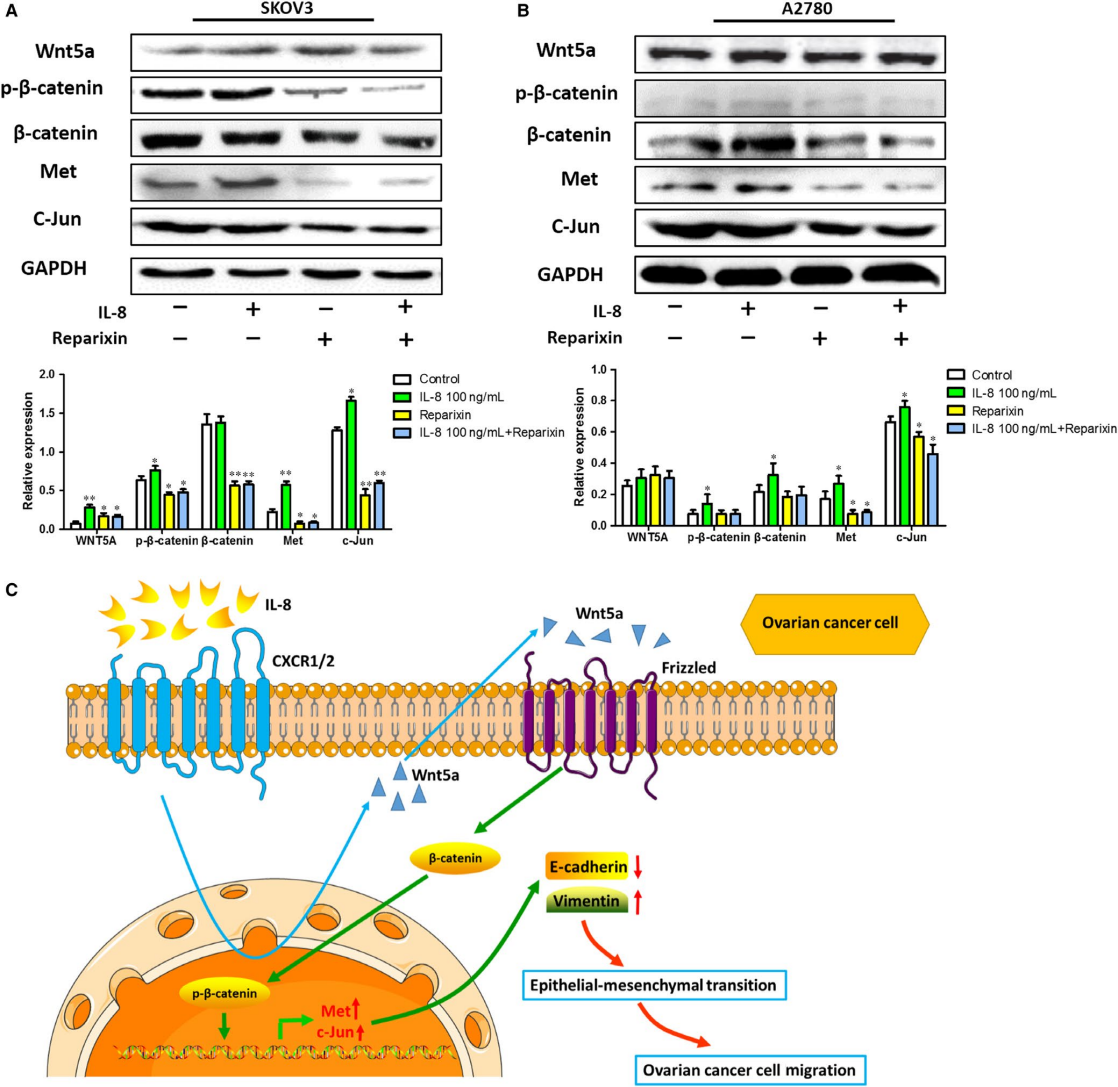
Expression of key proteins in Wnt signalling pathway in ovarian cancer cells. A, The expression levels of Wnt5a, p‐β‐catenin, β‐catenin, Met and C‐Jun in SKOV3 cells were quantified by image analysis of the Western blot bands. The expression of GAPDH in each group was taken as intrinsic controls, and relative expressions of each protein were calculated. B, The expression levels of Wnt5a, p‐β‐catenin, β‐catenin, Met and C‐Jun in A2780 cells were quantified by image analysis of the Western blot bands. Values represented the Mean ± SD from three independent experiments. *P < .05, **P < .01 vs Control. C, Diagram showed the effect of IL‐8 on the migration ability of ovarian cancer cells. IL‐8 up‐regulated the Wnt signalling ligand Wnt5a expression and subsequently activated a β‐catenin–dependent activation of Wnt pathway, which increased the expression of Wnt pathway cascade protein Met and c‐Jun, to finally induce a EMT process to promote the migration of ovarian cancer cells

To clearly understand the mechanisms of IL‐8 in ovarian cancer, we tested whether suppressing IL‐8 affected Wnt signalling pathway whose aberration played a crucial role in ovarian cancer progression. As shown in Figure 6, with the addition of IL‐8, Wnt5a, β‐catenin and p‐β‐catenin were elevated, the elevated p‐β‐catenin further promoted the expression of Met and C‐Jun. However, the expression of the key proteins in Wnt signalling pathway decreased in Reparixin group compared with control group, indicating that Reparixin weakened the promoting effect of endogenous IL‐8 on the activation of Wnt/β‐catenin pathway. Besides, the combined treatment of Reparixin and exogenous IL‐8 counteracted the promotion of IL‐8 on the activation of Wnt/β‐catenin pathway to some extent. These results strongly suggested that IL‐8 was involved in Wnt/β‑catenin signalling pathway and may elevate the signalling activity in ovarian cancer cells.

## 4 DISCUSSION

Ovarian cancer exhibited malignance and cause the highest mortality rate among all female reproductive system malignancies because of their strong invasive and metastatic abilities. As an inflammatory chemokine, IL‐8 was considered to stimulate tumour cell migration and proliferation and promote angiogenesis, which may eventually lead to tumour metastasis.^15^ This study identified the role for IL‐8 in ovarian cancer migration. First, we examined IL‐8, CXCR1 and CXCR2 expression in samples of ovarian serous cancer tissue and found the IL‐8, CXCR1 and CXCR2 were highly expressed in high‐grade ovarian serous cancer tissues than in low‐grade ovarian serous cancer tissues. Alternatively, patients with more advanced ovarian cancer (those with lower differentiation, distant metastasis and advanced FIGO stage) had increased tumoral IL‐8, CXCR1 and CXCR2 expression. Furthermore, CXCR2 was significantly related with poor overall survival of patients with ovarian cancer. Likewise, other studies revealed the similar result. Browne A found IL‐8, its receptors were expressed in ovarian cancer and there was a significant correlation between the expression and tumour stage and tumour type.^16^ Martins‐Filho A found patients with malignant ovarian cancer had higher IL‐8 levels compared to patients with benign neoplasms.^17^ Additionally, in evaluating the prognostic factors and IL‐8 levels of ovarian cancer, the increase in IL‐8 levels was found to be related to the negative prognostic factors of ovarian cancer.^18^ Moreover, inhibition of IL‐8 signalling may enhance the response to anticancer drug, such as platinum and Bortezomib.^19^, ^20^ Thus, to our knowledge, IL‐8, CXCR1 and CXCR2 may be correlated with the development and metastasis of ovarian cancer.

CXCR1 and CXCR2 were two G‐protein–coupled receptors which can be activated by IL‐8 or other chemokines, thus activating several intracellular signalling pathways.^21^ CXCR1 and CXCR2 were also found on the surface of the ovarian cancer cells and mounting evidence suggested that CXCR2 was the main receptor of IL‐8 in ovarian cancer cells,^22^, ^23^, ^24^ which was consist with our results of immunocytochemistry. To confirm the expression of CXCR1 and CXCR2 in ovarian cancer cells, we employed immunocytochemistry and found CXCR2 was mainly expressed in the ovarian cancer cells. Importantly, up‐regulation of CXCR2 had been reported to promote ovarian cancer progression and the high expression of CXCR2 was closely related with lower progression‐free survival and post‐progression survival, indicating the importance of CXCR2 in affecting ovarian cancer recurrence and metastasis.^23^, ^25^

To investigate the possible role of IL‐8 in ovarian cancer, we exogenously activated or inhibited IL‐8 effect in ovarian cancer cells using IL‐8 or Reparixin (inhibitor of both IL‐8 receptors CXCR1, CXCR2). Exogenous IL‐8 promoted ovarian cancer cell migration, while Reparixin reversed the effect of both endogenous and exogenous IL‐8. So how did the IL‐8 promote the migration of ovarian cancer? EMT was one of the most important steps of tumour metastasis, in which cancer cells more expressed mesenchymal markers rather than epithelial marker.^26^ EMT occurred under physiological conditions, but also a key mechanism required for malignancy. EMT led cancer cells to migrate and protected them from hostile environment during the dissemination process.^27^ The EMT‐associated markers E‐cadherin expression could reflect the ovarian cancer malignancy because E‐cadherin expression was associated with cancer invasion and metastasis.^28^ By inhibiting E‐cadherin expression and inducing EMT in ovarian cancer cells, the enhanced invasive and metastatic abilities of ovarian cancer cells were achieved.^29^ In our study, we found that IL‐8 led to an increased protein level of vimentin and a decreased protein level of E‐cadherin. Therefore, IL‐8 may regulate the ovarian cancer EMT to promote the ovarian cancer migration.

A variety of signalling pathways, including Wnt, TGF‐β and Notch, could trigger EMT. Although TGF‐β and Notch pathway could regulate the EMT, Wnt pathway was the most relevant signalling pathway with the IL‐8. IL‐8, as the up‐stream of Wnt/β‐Catenin, could induce the activation of Wnt pathway in cancer and reduce the degradation of β‐Catenin to induce cancer EMT and cancer metastasis.^30^, ^31^, ^32^ Additionally, the canonical Wnt/β‐catenin pathway had been proved to induce EMT in various cancer including ovarian cancer.^33^ As an important regulator of EMT, the Wnt signalling pathway played a crucial role in weakening the adhesion between tumour cells and enhancing the ability of tumour cells to invade.^34^, ^35^, ^36^ Thus, we selected several proteins which indicated the activation of Wnt/β‐catenin signalling pathway to identify the Wnt/β‐catenin signalling pathway. Wnt5a, a Wnt‐related protein, was proved to be up‐regulated in some solid tumours such as pancreatic cancer, skin cancer and gastric cancer.^37^, ^38^ As a Wnt ligand, Wnt5a could activate the Wnt/β‐catenin pathway in the presence of frizzled 4 (FZD4) and lipoprotein receptor‐related protein 5 (LRP5).^39^ Wnt5a could also regulate the activity of EMT, and Wnt5a‐overexpressing was found to elevate expression of vimentin and N‐cadherin and reduce expression of E‐cadherin.^40^, ^41^ In our study, the Wnt5a increased when under the stimulation of IL‐8, which indicated the activation effect of IL‐8 on the Wnt/β‐catenin signalling pathway. We also investigated the β‐catenin, a key active protein in Wnt/β‐catenin pathway, which formed adherens junctions together with E‐cadherin.^42^ As well known, β‐catenin is the central player of the Wnt signalling pathway and the activation of the β‐catenin signalling is related to the initiation of EMT.^43^, ^44^ In our study, we found that β‐catenin and functional β‐catenin expression changed in response to IL‐8 or Reparixin treatment, which indicated the IL‐8 effects on the key activity of the Wnt/β‐catenin pathway. Met, a cell membrane receptor tyrosine kinase, is also a recognized Wnt target gene and up‐regulated by the regulation of Wnt/β‐catenin signalling cascade.^45^, ^46^ On the other hand, Met also plays an important role in the pathogenesis of ovarian cancer.^47^ So it is meaningful to examine the Met expression in the ovarian cancer when we research the Wnt/β‐catenin pathway. As for c‐Jun, it is a β‐Catenin target gene, plays a positive regulatory role in EMT of various tumour cells and inhibits the expression of c‐Jun, or its associated pathways would reduce cancer cell EMT process.^48^, ^49^, ^50^, ^51^ Considering that c‐Jun up‐regulation in ovarian cancer cells may lead to induction of EMT, we checked this protein to verify the regulatory role of Wnt/β‐catenin pathway in induction of EMT. In the results, with the addition of IL‐8, Wnt5a, β‐catenin and p‐β‐catenin were elevated, the elevated p‐β‐catenin further promoted the expression of Met and C‐Jun. However, following inhibition of IL‐8 effect, Wnt5a, p‐β‐catenin, β‐catenin, Met and C‐Jun in the Wnt/β‐catenin pathway were decreased. These results strongly suggested that IL‐8 was involved in Wnt/β catenin signalling pathway and may elevate the signalling activity in ovarian cancer cells. Thus, from our point of view, IL‐8 up‐regulated the Wnt signalling ligand Wnt5a expression, and subsequently activated a β‐catenin–dependent activation of Wnt pathway, which increased the expression of Wnt pathway cascade protein Met and c‐Jun, to finally induce a EMT process to promote the migration of ovarian cancer cells.

## 5 CONCLUSION

Collectively, this study demonstrated that IL‐8 and IL‐8 receptors CXCR1 and CXCR2 were up‐regulated in advanced ovarian serous cancer tissues. Furthermore, we revealed that IL‐8 enhanced ovarian cancer by initiating an EMT program to promote cancer cell migration, and we also found that the Wnt/β‐catenin pathway may be involved in mediating the EMT process. These results suggested that IL‐8 and IL‐8 receptors CXCR1 and CXCR2 were potential biomarkers and therapeutic targets for ovarian cancer.

## CONFLICT OF INTEREST

The authors declare that they have no competing interests.

## AUTHOR CONTRIBUTION

Jirui Wen, Zhiwei Zhao, Yali Miao and Jiang Wu contributed to study concept and design; Liwei Huang and Ling Wang contributed to analysis and interpretation of the data; Jirui Wen, Zhiwei Zhao, Yali Miao and Jiang Wu contributed to drafting of the manuscript; Yali Miao and Jiang Wu contributed to revision of the article.

## Data Availability

The data are available from the corresponding author on reasonable request.
